# Diaportheone A Analogues Instigate a Neuroprotective Effect by Protecting Neuroblastoma SH-SY5Y Cells from Oxidative Stress

**DOI:** 10.3390/biology10030199

**Published:** 2021-03-05

**Authors:** Mario A. Tan, Elena Zakharova, Seong Soo A. An

**Affiliations:** 1 Department of Bionano Technology, Bionano Research Institute, Gachon University, 1342 Seongnam-daero, Sujung-gu, Seongnam-si 461-701, Korea; 2 College of Science and Research Center for the Natural and Applied Sciences, University of Santo Tomas, Espana, Manila 1015, Philippines; 3 Department of Chemistry and Biochemistry, University of Bern, Freiestrasse 3, 3012 Bern, Switzerland; elena.zakharova@dcb.unibe.ch

**Keywords:** Alzheimer’s disease, amyloid-*beta*, chromone, molecular docking, oxidative stress

## Abstract

**Simple Summary:**

Alzheimer’s disease is a progressive neurodegenerative illness affecting mostly elderly people. Preventing the neurotoxicity caused by the formation of amyloid-*beta* plaques and oxidative stress is one of the key strategies to minimize the effects of this disease. Hence, our study aims to search for compounds which may exhibit neuroprotective potential using the human neuroblastoma SH-SY5Y cells induced with oxidative stress as our cellular model. Our library of compounds showed that diaportheones A1 and A2 protected the formation of amyloid-*beta* plaques using the ThT assay and exhibited neuroprotective effects in damaged SH-SY5Y cells. The preventive effect of the compounds on the aggregation of amyloid-*beta* was also shown by molecular modelling. Thus, diaportheones A1 and A2 could be potential compounds for further studies against Alzheimer’s disease.

**Abstract:**

Alzheimer’s disease (AD) remains an incurable neurodegenerative illness. Oxidative stress resulting in the formation of reactive oxygen species (ROS) and the abnormal deposition of amyloid-*beta* (Aβ) are the major pathological hallmarks associated with AD. In search for small molecules targeting multiple pathways of AD and of no known molecular targets, the neuroprotective effects of the synthetic chromones diaportheone A1 and diaportheone A2, analogues of the natural product diaportheone A, were investigated. Chromones are heterocyclic compounds bearing the benzoannelated γ-pyrone moiety and were regarded as an important class of organic molecules due to their diverse pharmacological activities. The influence of the compounds on the inhibition of Aβ aggregation was determined by Thioflavin T (ThT) assay, and the cell viability, ROS, and mitochondrial membrane potential were evaluated with human neuroblastoma SH-SY5Y cells. Results showed that both compounds inhibited the Aβ aggregation at 80.41% and 73.68% for diaportheone A1 and diaportheone A2, respectively. Increased cell viabilities were observed from the protection by both compounds using Aβ- or H_2_O_2_-induced SH-SY5Y cells. Both compounds also reduced the intracellular ROS level in Aβ- or H_2_O_2_-induced SH-SY5Y cells at 10 and 20 μM concentrations, and increased the mitochondrial membrane potentials in Aβ-induced SH-SY5Y cells at 20 μM concentration. Molecular docking experiments using the Aβ protein models 2MXU and 2BEG also indicated a good agreement with the experimental data. The results demonstrated for the first time the oxidative stress effects associated with the chromones diaportheone A1 and diaportheone A2 as potential neuroprotective therapeutic agents against AD.

## 1. Introduction

Alzheimer’s disease (AD) remains an incurable neurodegenerative illness among elderly people and is characterized by memory loss, dementia, and the progressive deterioration of cognition and language skills [1]. Many research investigations are continuously revealing the vast interconnected complex systems with genetic and biochemical factors for better understanding its pathogenicity [2,3,4,5]. The worldwide population estimates that around 47 million elderly people are affected by the disease, with the number rising to 131 million in 2050 [6]. Although there is no cure yet for AD, four drugs targeting acetylcholinesterase and one drug targeting *N*-methyl-D-aspartate receptor are currently available to reduce the symptoms of AD patients [7]. Strategic treatments in the early stage of AD detection are also proven to be beneficial [8].

Oxidative stress is usually involved in the production of high level of reactive oxygen species (ROS) and reactive nitrogen species, resulting in the significant reduction of the cellular antioxidant defenses [9]. In general, ROS contributes to biological processes such as cellular metabolic regulations and are in equilibrium with the endogenous antioxidant system [10]. However, an imbalance in the system producing more ROS leads to oxidative damage in the cellular structure and cell death [10]. Hence, oxidative stress is associated as a leading cause of neurodegenerative diseases such as AD, Parkinson’s disease, amyotrophic lateral sclerosis (ALS), and even stroke [10]. Oxidative stress resulting in the formation of ROS and the abnormal deposition of amyloid-*beta* (Aβ) are the major pathological hallmarks associated with AD [9]. The association of the oxidative stress and the formation of Aβ aggregates leading to oxidative damages to neurons in cell culture, to AD animal models, and the human brain were well documented [11,12,13,14]. Hence, the discovery of drugs multi-targeting the inhibition of Aβ aggregation and oxidative stress is eagerly warranted.

Due to the chemical diversity of small molecules, whether synthetically derived or isolated from natural products, many compounds were subjected to intense AD research for potential leads in the field of drug discovery and development. Various chemical scaffolds belonging to the alkaloids, phenolics, terpenoids, flavonoids, glucosides, and saponins exhibit protective effects using in vitro and in vivo models [15]. The plant-derived alkaloids galantamine and rivastigmine are examples of approved anti-AD drugs targeting the acetylcholinesterase (AChE) pathway [7]. Small molecules reported in the literature demonstrated their potential for anti-AD drug discovery as inhibitors of neuroinflammation, tau protein hyperphosphorylation [16], neuroprotective effects against Aβ toxicity, aggregation, or oligomerization [7,17], or the reduction of oxidative stress [18]. However, due to the complexity of the AD, the search for small molecules targeting multiple pathways should be essential as an alternative mechanism to potential AD therapy [7]. Hence, this study examined the neuroprotective ability of the synthetic chromone molecules diaportheone A1 and diaportheone A2 in vitro, using the neuroblastoma SH-SY5Y cells. Literature search indicated no known molecular targets or biological activities associated with these compounds. Neuroblastoma SH-SY5Y cells were induced with Aβ_1-42_ or H_2_O_2_ to determine the neuroprotective effects, and the Thioflavin T (ThT) assay was used to assess their inhibition of Aβ aggregation.

## 2. Materials and Methods

### 2.1. Thioflavin T (ThT) Assay

The procedure for the ThT assay was previously reported [2,19,20]. Briefly, Aβ_1-42_ (Aggresure™ (AnaSpec) Fremont, CA, USA) was dissolved in phosphate buffered saline (PBS) and incubated with or without the compounds or phenol red (positive control) at 37 °C for 24 h. ThT solution was added and incubated for 15 min. Fluorescence signal (Ex 450 nm; Em 510 nm) was measured using a PerkinElmer Victor-3® multi-plate reader (PerkinElmer, Waltham, MA, USA). The percentage of aggregation inhibition was calculated using the following equation: [(1−*I*_Fi_/*I*_Fc_) × 100%], where *I*_Fi_ and *I*_Fc_ are the fluorescence absorbance with and without the inhibitors, respectively, after subtracting the background fluorescence of the ThT solution.

### 2.2. Cell Culture

Human neuroblastoma SH-SY5Y cells (American Type Culture Collection, ATCC CRL-2266) (Manassas, VA, USA) were maintained in Dulbecco’s modified eagle media (DMEM) supplemented with 10% fetal bovine serum (FBS), 1% kanamycin, and 1% penicillin at 37 °C and 5% CO_2_, and passaged once per week. Experiments were performed at 80–90% cell confluence.

### 2.3. Cell Cytotoxicity

Cell viability measurement was performed using the ATP luminescence assay as previously described [19,20]. SH-SY5Y cells were counted by Eve cell counter (NanoEntek, Inc., Seoul, Korea), and 2 × 10^4^ cells/well density were sub-cultured in 96-well plate and incubated for 24 h. After incubation, cells were treated with the compounds for 24 h. The media were removed, cells were washed with PBS, fresh media were added, and incubated for another 30 min. Then, CellTiter-Glo^®^ (Promega, Madison, WI, USA) luminescent reagent was added and the luminescence was read on a PerkinElmer Victor-3^®^ multi-plate reader. Data were analyzed and the % cell viability was expressed relative to the control.

### 2.4. Neuroprotective Activity Assay

Determination of the neuroprotective activity in Aβ or H_2_O_2_-induced SH-SY5Y cytotoxicity was performed as previously described [21,22] and evaluated by the ATP luminescence assay as described above. Neuroblastoma SH-SY5Y cells were seeded in a 96-well plate at 2 × 10^4^ cells/well and incubated for 24 h. After stabilization, cells were pre-treated with the compounds for 6 h before incubation with Aβ (5 μM) or H_2_O_2_ (100 μM) for 24 h. A solvent control (untreated control cells), Aβ or H_2_O_2_ alone, and the compounds alone treatments were also included. After incubation, the % cell viability was determined in triplicate experiments.

### 2.5. Measurement of Reactive Oxygen Species (ROS)

Intracellular ROS level measurement was performed using the 2′,7′-dichlorodihydrofluorescein diacetate (H_2_DCFDA) (Sigma Aldrich, St. Louis, MO, USA) staining method, as previously described [21,23]. After 24 h acclimatization, SH-SY5Y (2 × 10^4^ cells/wells) cells were pre-treated with the compounds for 2 h before incubation, with 5 μM Aβ for 24 h or 100 μM H_2_O_2_ for 4 h. Cells were then treated with 25 μM H_2_DCFDA and incubated for 2 h in the dark at 37 °C. Fluorescence intensity (Ex 495 nm, Em 520 nm) was measured in a microplate reader. The ROS level was calculated as a percentage of the untreated control cells (100%) in triplicate measurements.

### 2.6. Mitochondrial Membrane Potential (ΔΨm) Assay

Measurement of the ΔΨm was performed using the tetramethylrhodamine, methyl ester (TMRE) (Abcam TMRE mitochondrial membrane kit) staining method as previously described [24] and following the manufacturer’s protocol. SH-SY5Y (2 × 10^4^ cells/well) cells were pre-treated with the compounds for 2 h, and incubated together with 5 μM Aβ for 24 h. After treatment, 1 μM TMRE staining solution was added and incubated at 37 °C for 1 h. The fluorescence (Ex 549 nm, Em 575 nm) was read in a microplate reader. The ΔΨm was calculated as a percentage of the untreated control cells (100%) in triplicate measurements.

### 2.7. Molecular Docking

The software AutoDock Tools (La Jolla, CA, USA) (version 1.5.6) [25] was used to perform a blind docking of diaportheone A1 and diaportheone A2 into ten protein models of the recently resolved NMR structures of Aβ42 fiber (PDB codes: 2BEG and 2MXU) [26,27] as follows. Polar hydrogens and Kolman charges were added to the receptor. The Lamarckian genetic algorithm (GA) was used for ligand conformation search with the following parameters: number of GA runs 50; population size 300; the other parameters are default. Since the molecule has only two rotatable bonds, the applied procedure is reliable to find putative binding sites.

Fifty docking solutions for each ligand in each protein model were generated and energetically scored. Thus, one thousand (2 × 10 × 50) docking solutions for each protein molecule were analyzed. All the results were evaluated to explore binding modes and their energies for different sites. The binding energy maps were plotted with PyMOL (version 1.7.4, The PyMOL Molecular Graphics System; Schrödinger, LLC: New York, NY, USA).

### 2.8. Statistical Analysis

Data are reported as the mean ± SD of at least three experiments. Statistical analysis was determined by one-way ANOVA followed by Tukey’s HSD post-hoc test (GraphPad Prism 5 software package, version 5.02, GraphPad Software Inc., San Diego, CA, USA). Statistical significance was considered at *p* < 0.05.

## 3. Results

**Figure 1:**
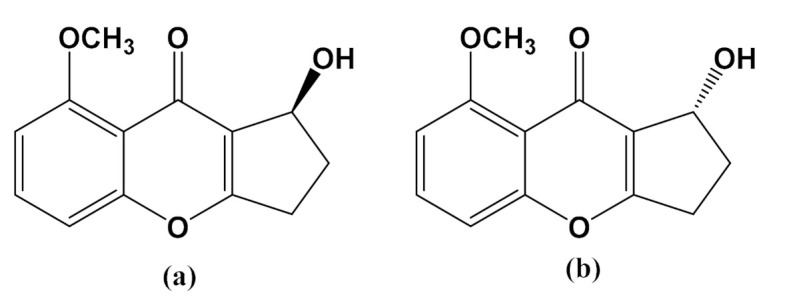
Structure of (**a**) Diaportheone A1 and (**b**) Diaportheone A2.

The current research is part of our on-going study to search for biologically active compounds against AD using our library of plant-derived natural products and synthetic compounds. Prioritization was done on new entities with no reported biological activities. In the present study, diaportheone A1 and diaportheone A2 (Figure 1) are synthetic scaffolds with no reported molecular targets. We tested their potential as Aβ aggregation inhibitors and neuroprotective agents using in vitro and molecular docking.

### 3.1. Effects of Diaportheone A1 and Diaportheone A2 on Thioflavin T (ThT) Assay

The effects of the compounds (Figure 1) to inhibit the Aβ aggregation was evaluated using the ThT fluorescence assay. The measured fluorescence in the assay was the result of the binding of the ThT to the Aβ aggregates. Aβ (10 μM) solutions were incubated with the compounds at 5 and 50 μM concentrations for 24 h. Diaportheone A1 (*S*-1-hydroxy-8-methoxy-2,3-dihydrocyclopenta[*b*]chromen-9(1*H*)-one) exhibited 80.41% ± 1.40 inhibition at 50 μM, and 34.75% ± 2.5 inhibition at 5 μM concentration. Diaportheone A2 (*R*-1-hydroxy-8-methoxy-2,3-dihydrocyclopenta[*b*]chromen-9(1*H*)-one) showed 73.68% ± 1.70 (at 50 μM) and 35.21% ± 2.80 (5 μM) inhibitions. All percentage inhibitions (Table 1) exhibited significant difference (*p* < 0.05), with phenol red as the positive control [28,29] (65.78% ± 2.97 at 50 μM).

### 3.2. Cytotoxicity of Diaportheone A1 and Diaportheone A2

**Figure 2:**
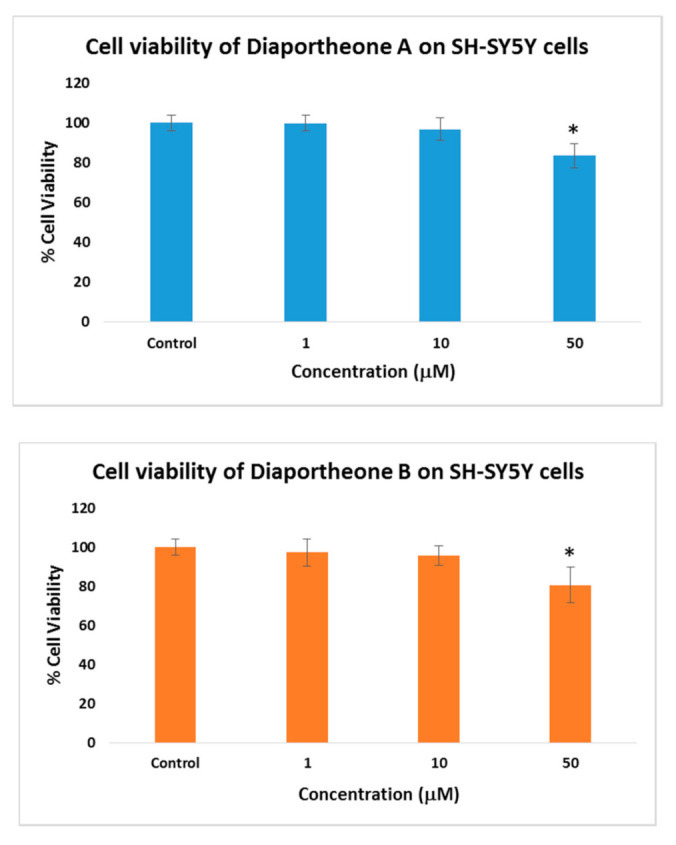
Cytotoxic effects of diaportheone A1 and diaportheone A2 on the neuroblastoma SH-SY5Y cells measured using the ATP assay. The cells were treated for 24 h with varying compound concentrations. The cell viability is reported as percentage of the control group (0 μM set as 100%). All data are presented as mean ± SEM (*n* = 3). Significant difference (*) (*p* < 0.05) using one-way ANOVA followed by Tukey’s test was observed to the % cell viability vs the control group.

The cell viability of the compounds was evaluated at different concentrations (1, 10, 50 μM) after 24 h of treatment (Figure 2) using the ATP luminescence assay. Both compounds did not show any significant cytotoxicity when compared to the control cells. Both the 1 and 10 μM concentrations exhibited > 95% cell viability. At the highest concentration (50 μM), 83% cell viability for diaportheone A1 and 80% cell viability for diaportheone A2 were observed, which exhibited statistically significant differences (*p* < 0.05) when compared to the control cells. Hence, to minimize the inhibition effect of the compounds against the neuroblastoma cells, the succeeding neuroprotective experiments used 1, 10 and 20 μM concentrations.

### 3.3. Neuroprotective Effects of Diaportheone A1 and Diaportheone A2

**Figure 3:**
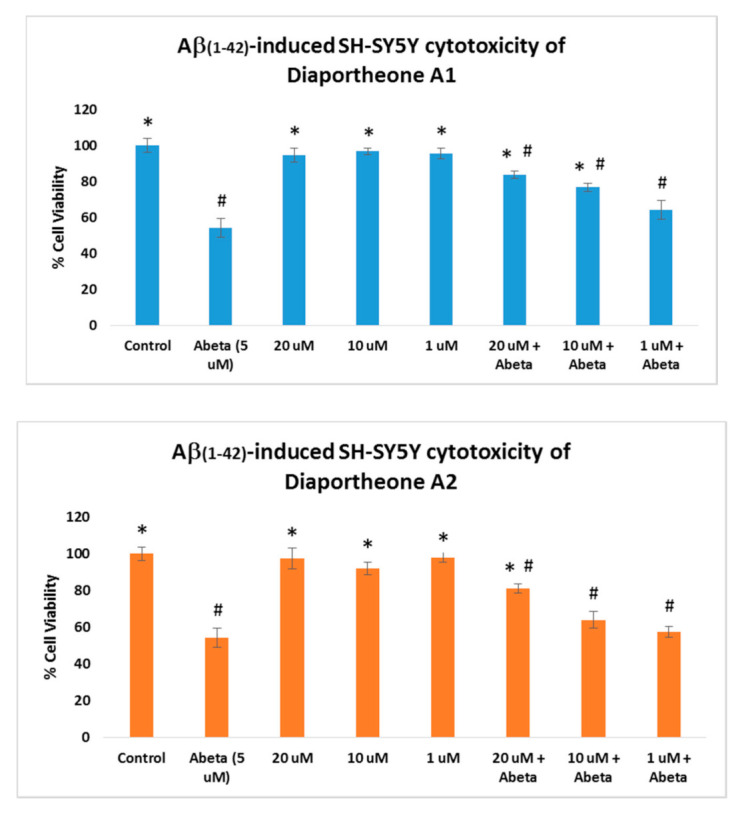
Neuroprotective effects of diaportheone A1 and diaportheone A2 on Aβ-induced neuroblastoma SH-SY5Y cells. The SH-SY5Y cells with the compounds only were incubated for 24 h. For the Aβ-treated cells, the SH-SY5Y cells were incubated with the compounds for 6 h, followed by Aβ-treatment (5 μM) for 24 h. Results indicate % cell viability vs. the control cells (mean ± SEM of triplicate trials). Statistical difference (*p* < 0.05) using one-way ANOVA followed by Tukey’s test of the % cell viability versus the Aβ (Abeta at 5 μM)-treated alone group (*) or the control group (#).

The neuroprotective effects of the compounds were evaluated using Aβ-induced and H_2_O_2_-induced neuroblastoma SH-SY5Y cells. For the Aβ-induced treatment (Figure 3), the cells were pretreated with the compounds for 6 h followed by treatment with the Aβ (5 μM) for 24 h. Similarly, the cell viability was also compared to the cells treated only with the compounds for 24 h. As presented in Figure 3, the cells treated only with the compounds at 20, 10 and 1 μM concentrations did not show cytotoxic effects (cell viability > 95%) when compared to the control cells. The cells treated with Aβ only (5 μM) showed a 54.41% (±5.1) cell viability. The SH-SY5Y cells pre-treated with the compounds for 6 h, followed by treatment with Aβ (5 μM) for 24 h, exhibited significant increases in the cell viability (*p* < 0.05) at 10 μM (76.81% ± 2.31) and 20 μM (83.79% ± 2.02) concentrations for diaportheone A1, and 20 μM (81.05% ± 2.41) concentration for diaportheone A2 in comparison to the Aβ-treated alone cells. None of the compounds showed neuroprotective effects (*p* < 0.05) using the 1 μM concentration.

**Figure 4:**
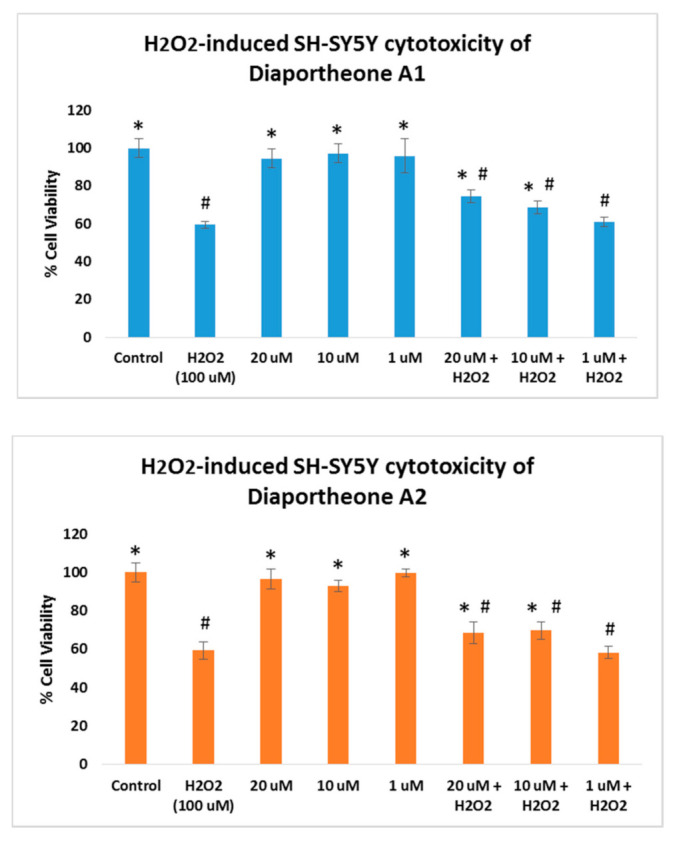
Neuroprotective effects of diaportheone A1 and diaportheone A2 on H_2_O_2_-induced neuroblastoma SH-SY5Y cells. The SH-SY5Y cells treated with the compounds only were incubated for 24 h. For the H_2_O_2_-treated cells, the SH-SY5Y cells were incubated with the compounds for 6 h, followed by H_2_O_2_-treatment (100 μM) for 24 h. Results indicate % cell viability vs. the control cells (mean ± SEM, *n* = 3). Statistical difference (*p* < 0.05) using one-way ANOVA followed by Tukey’s test of the % cell viability versus the H_2_O_2_-treated (100 μM) alone group (*) or the control group (#).

The neuroprotective effects of the compounds on oxidative stress were also explored by treating the SH-SY5Y cells with 100 μM H_2_O_2_. As shown in Figure 4, SH-SY5Y cells treated only with the compounds at 1, 10, and 20 μM concentrations for 24 h did not exhibit cytotoxicity (cell viability > 95%). In a parallel experiment, SH-SY5Y cells were pretreated with the compounds for 6 h, followed by H_2_O_2_ treatment for 24 h. The H_2_O_2_-alone treated cells for 24 h showed 59.43% (±2.01) cell viability. When compared to the SH-SY5Y cells treated both with the compounds and H_2_O_2_, the induced cytotoxicity generated by the H_2_O_2_ was attenuated by both compounds at 10 μM and 20 μM concentrations. Diaportheone A1 showed 68.73% ± 3.21 (at 10 μM) and 74.62% ± 3.42 (at 20 μM), while diaportheone A2 gave 69.75% ± 4.51 (at 10 μM) and 68.73% ± 5.33 (at 20 μM). No neuroprotective effect was observed for both compounds at 1 μM. The underlying mechanism involved in the neuroprotective effects was investigated utilizing the measurement of the ROS and the mitochondrial membrane potential.

### 3.4. Effects on Intracellular Reactive Oxygen Species (ROS) Production

**Figure 5:**
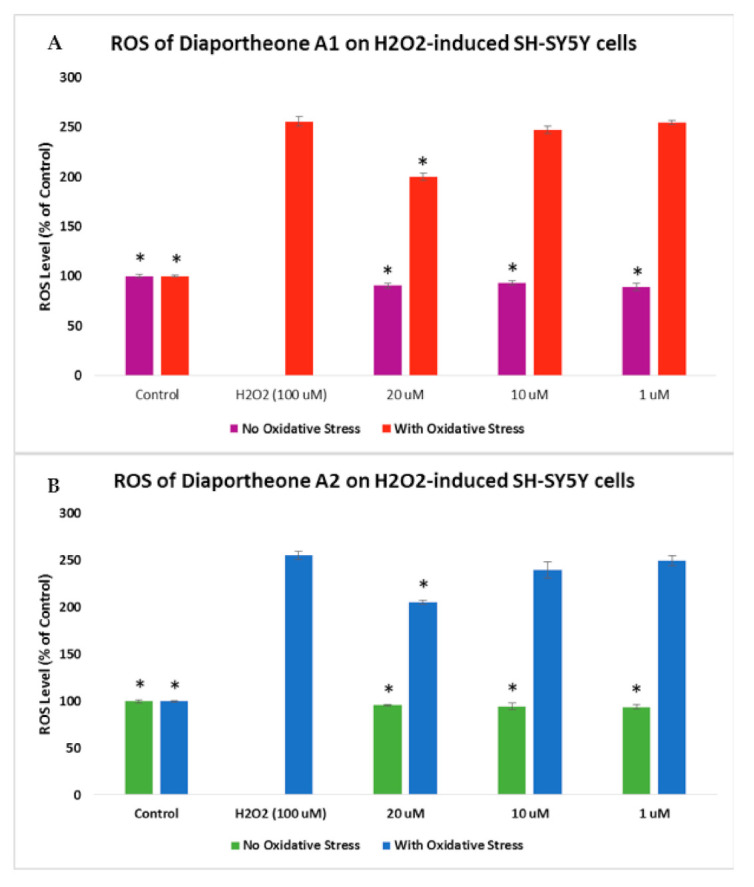
Effects of diaportheone A1 (**A**) and diaportheone A2 (**B**) on H_2_O_2_-induced intracellular ROS accumulation. The level of ROS in neuroblastoma SH-SY5Y cells was evaluated using 2′,7′-dichlorodihydrofluorescein diacetate (H_2_DCFDA) reagent. SH-SY5Y cells were pretreated with the compounds for 2 h, followed by treatment with 100 μM H_2_O_2_ for 4 h. No Oxidative Stress indicates treatment of the SH-SY5Y cells only with the compounds. The intracellular ROS level (% of the control cells) was expressed as the mean ± SEM (*n* = 3). The (*) represents statistical difference (*p* < 0.05) of the % ROS versus the H_2_O_2_-treated alone group using one-way ANOVA followed by Tukey’s test.

The level of ROS generation in Aβ- or H_2_O_2_-induced SH-SY5Y cells was evaluated using 2′,7′-dichlorodihydrofluorescein diacetate (H_2_DCFDA) reagent. In Figure 5, H_2_O_2_ (100 μM) was used in the generation of intracellular ROS in the SH-SY5Y cells. Initially, cells were pretreated with compounds for 2 h before incubating with the H_2_O_2_ for 4 h. The reduction in the incubation time with H_2_O_2_ is a result of the observed % cell viability (Figure 4), which also caused a decrease of fluorescence [21]. The SY-SY5Y cells treated with H_2_O_2_ only generated 255.78% ± 4.52 level of ROS. Treating the SH-SY5Y cells with 20 μM of the compounds showed a significant reduction (*p* < 0.05) in the ROS level, with 199.67% ± 3.58 for diaportheone A1 (Figure 5A) and 205.53% ± 2.34 for diaportheone A2 (Figure 5B) when compared to the H_2_O_2_-alone cells. At 10 μM and 1 μM concentrations, the level of ROS is comparable (*p* < 0.05) to the H_2_O_2_-alone cells. The intracellular ROS without H_2_O_2_ treatment (no oxidative stress) was also evaluated by treating the SH-SY5Y cells with the compounds for 6h. Results indicated in the no oxidative stress group showed comparable effects to the control cells.

**Figure 6:**
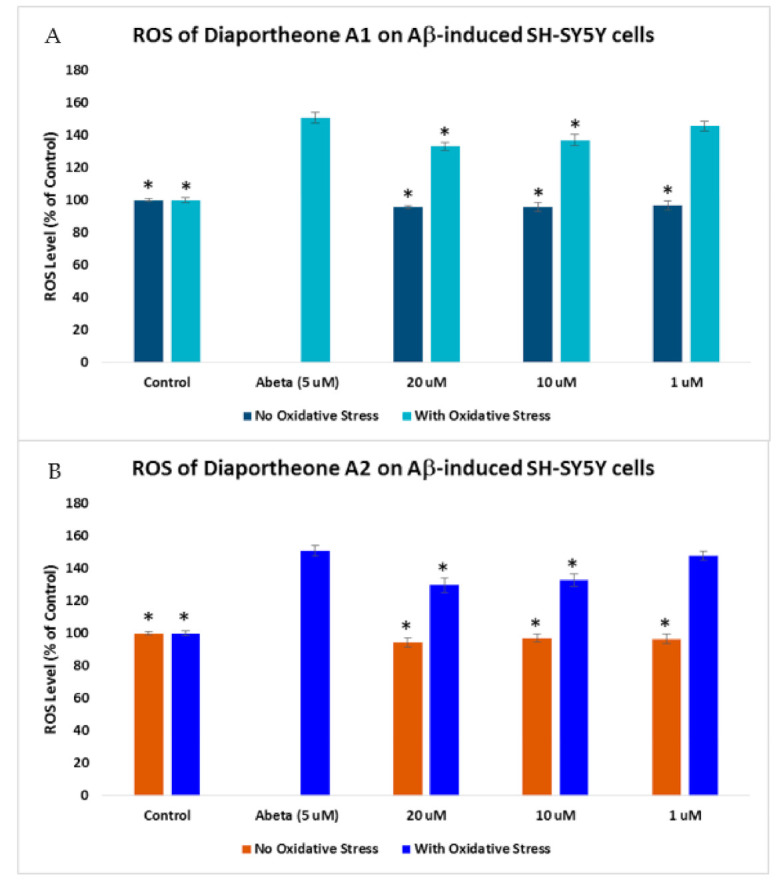
Effects of diaportheone A1 (**A**) and diaportheone A2 (**B**) on Aβ-induced intracellular ROS accumulation. The level of ROS in neuroblastoma SH-SY5Y cells was evaluated using 2′,7′-dichlorodihydrofluorescein diacetate (H_2_DCFDA) reagent. SH-SY5Y cells were pretreated with the compounds for 2 h, followed by treatment with 5 μM Aβ (Abeta) for 24 h. No Oxidative Stress indicates treatment of the SH-SY5Y cells only with the compounds. The intracellular ROS level (% of the control cells) was expressed as the mean ± SEM (*n* = 3). The (*) represents statistical difference (*p* < 0.05) of the % ROS versus the Abeta-treated alone group using one-way ANOVA followed by Tukey’s test.

The effect of the compounds on the ROS generation in Aβ-induced SH-SY5Y cells was also evaluated. The cells were pretreated with the compounds for 2 h before incubating with 5 μM Aβ for 24 h [30]. As shown in Figure 6, a 150.79% ± 3.44 ROS level was generated when the cells were treated only with Aβ for 24 h. When the SH-SY5Y cells were pretreated for 2 h with the compounds before Aβ exposure for 24 h, a significant decrease (*p* < 0.05) in the ROS level was observed at 10 μM (136.94% ± 3.41) and 20 μM (133.04% ± 2.65) for diaportheone A1 (Figure 6A), and at 10 μM (132.74% ± 3.59) and 20 μM (129.67% ± 4.52) for diaportheone A2 (Figure 6B).

### 3.5. Effects on Mitochondrial Membrane Potential

**Figure 7:**
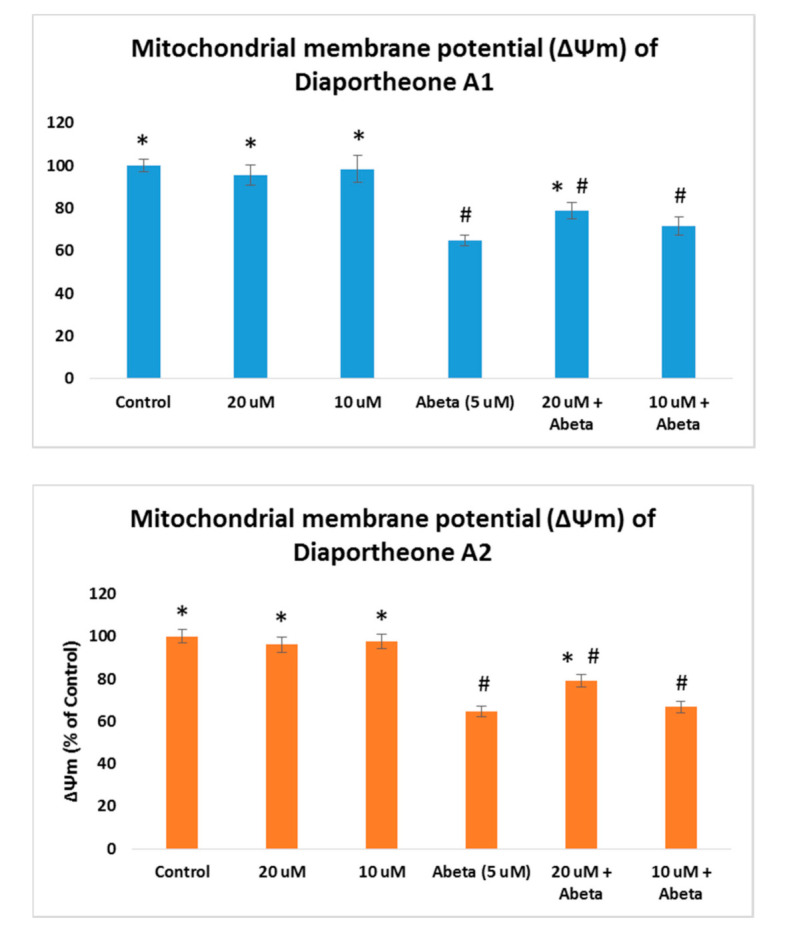
Effects on the mitochondrial membrane potential (ΔΨm) of diaportheone A1 and diaportheone A2. ΔΨm were evaluated in Aβ-treated (5 μM) SH-SY5Y cells using the tetramethylrhodamine, methyl ester (TMRE) assay. Cells were pretreated with the compounds for 2 h, followed by Aβ treatment for 24 h. The ΔΨm (% of the control cells) was expressed as the mean ± SEM (*n* = 3). Statistical difference (*p* < 0.05) of the ΔΨm versus the Abeta-treated alone group (*) or the control group (#) using one-way ANOVA followed by Tukey’s test.

As the increase of ROS accumulation is the result of mitochondrial dysfunction, the effects of the compounds on mitochondrial membrane potential (ΔΨm) were evaluated using the TMRE staining assay. The ΔΨm was conducted in Aβ-induced SH-SY5Y cells as both compounds showed ROS protection at 10 μM and 20 μM concentrations. The SH-SY5Y cells were pretreated with the compounds for 2 h, followed by Aβ (5 μM) for 24 h. As presented in Figure 7, a significant increase (*p* < 0.05) when compared to the Aβ-treated only (64.79% ± 2.56) cells was observed at 20 μM concentration for diaportheone A1 (79.02% ± 3.85) and diaportheone A2 (78.95% ± 3.01). None of the compounds showed a significant effect at 10 μM on this parameter. SH-SY5Y cells treated only with the compounds gave a similar ΔΨm level with the control cells.

### 3.6. Molecular Docking

**Figure 8:**
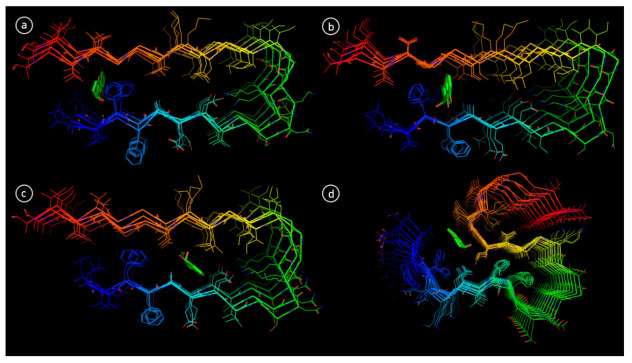
Binding modes of diaportheone A1 in Aβ fibril revealed by molecular docking; (**a**) 2BEG, site 1, model 1; (**b**) 2BEG, site 2, model 5; (**c**) 2BEG, site 3, model 8; (**d**) 2MXU, model 1.

In order to explore the structural basis of Aβ aggregation inhibition by diaportheone A1 and diaportheone A2, molecular docking was performed. Two protein NMR structures of Aβ_1-42_, 2BEG (pentamer) and 2MXU (dodecamer) were used for molecular docking, as they have different oligomeric composition [26,27]. Ten available conformational NMR models for both 2BEG and 2MXU were examined for Aβ_1-42_-ligand interactions. Having the reference data from other work [31], three main sites were found, the 2BEG conformer (Figure 8a–c).

Site 1 is formed by residues Leu17, Val18, Phe19, Gly38, Val39 and presented in 6 out of 10 NMR models (Models 1, 2, 3, 6, 8, 10 in Table 2). Site 2 is composed of the residues Phe19, Phe20 and Gly37 and relevant only for models 7, 8 and 9. Site 3 is shaped by residues Ala21, Glu22 and Asp23 (applicable for models 4, 5 and 7). By comparison of the binding affinities of diaportheone A1 and diaportheone A2, the docking results of each model showed moderate differences (Table 2). In the position of site 1, diaportheone A1 is more favorable for most of the models except for models 1 and 3. At Site 2, diaportheone A1 and diaportheone A2 have comparable binding affinities. At Site 3, models 4 and 7 showed the privilege of diaportheone A1 (−6.8 and −8.1 kcal/mol compared to −6.6 and −7.4 kcal/mol for diaportheone A2). Model 5 showed a slight gain of diaportheone A2 (−8.8 kcal/mol and for diaportheone A1 −8.7 kcal/mol).

Similarly, the docking performed for 2MXU with 10 different NMR models revealed few binding sites. In this case, only one and the most common site was extracted and have already been previously reported [32]. This site is delimited by residues Ile32, Gly33, Leu34 and Gln15 (Figure 8d). Docking results for 2MXU demonstrated more consistent trend, with only model 7 not showing any binding at chosen site. As exhibited in Table 2, for all the models except 4, diaportheone A1 showed better binding affinity when compared to diaportheone A2.

## 4. Discussion

Chromones are oxygen-bearing heterocyclic compounds containing the benzoannelated γ-pyrone moiety. They comprised a group of chemically varied structures that are isolated in nature. Pharmacological activities identified with the chromones included anti-inflammatory, antimicrobial, antitumor, anti-diabetic, diuretics, hypoglycemic, hypolipidemic, antioxidant, and neurodegenerative enzyme inhibitors [33]. The chromone structure is an excellent target in medicinal chemistry due to their structural diversity and synthetic accessibility [29]. The synthetic and natural chromone compounds with reported anti-neurodegenerative effects were limited to acetylcholinesterase (AChE), β-secretase-1 (BACE-1), and monoamine oxidase B (MAO-B) inhibitors and serotonin receptors [33,34].

The present study of synthetic chromones diaportheone A1 and diaportheone A2 presented information on their inhibitory activity against Aβ aggregation and neuroprotective effects against the oxidative stressors Aβ- or H_2_O_2_-induced human neuroblastoma SH-SY5Y cells. Both chromones were synthetic intermediates as racemic mixture in the total synthesis of diaportheone A [35]. They were resolved by chiral supercritical fluid chromatography in >99% enantiometic excess and their absolute configuration was unambiguously determined by X-ray crystallography [35]. Structurally, the methoxy group (−OCH_3_) in diaportheones A1 and A2 is replaced by a hydroxyl group in diaportheone A. To the best of our knowledge, this is the first report on the biological activities of these chromones.

The development of an effective drug for the treatment of AD was challenging, even after many investigations with diverse isolated natural products and synthetic compounds [36]. Many complex biochemical and metabolic pathways were intertwined in AD progressions and pathogenicity. Being a multifaceted illness, a drug for targeting multiple pathological hallmarks of AD should be envisioned as a possible avenue for the treatment of the disease, instead of concentrating on only one pathological target [24].

In this study, both compounds were able to inhibit the aggregation of Aβ as evaluated in the ThT assay. To further explore their potential, the neuroprotective effects in damaged neuroblastoma SH-SY5Y cells were investigated. In the Aβ-induced SH-SY5Y cells or H_2_O_2_-treated SY-SY5Y cells, an increase in the cell viability was observed, thereby avoiding the cytotoxic effects in the SH-SY5Y cells of either Aβ or H_2_O_2_. A possible mechanism involves the reduction of the oxidative stress. Oxidative stress is a known characteristic of chronic and acute illnesses, including neurodegenerative diseases. The presence of oxidative stress would also lead to mitochondrial dysfunctions as a result of an increased ROS. As a consequence, the buildup of oxidative damages leads to neuronal death causing age-related illnesses including AD. The reductions of ROS generation in the SH-SY5Y cells after H_2_O_2_ or Aβ treatments were successfully manifested by diaportheone A1 and diaportheone A2. Results also indicated that both compounds decreased the mitochondrial dysfunctions by elevating the mitochondrial membrane potential in Aβ-induced SH-SY5Y cells.

Molecular docking studies of Aβ with diaportheone A1 and diaportheone A2 suggested that these scaffolds can interact with Aβ oligomers, thus, presumably, blocking aggregation. The protein model 2MXU demonstrated increased affinity to diaportheone A1 compared to diaportheone A2, which is in good agreement with experimental data.

Taken together, the chromones diaportheone A1 and diaportheone A2 could be promising compounds for AD treatment. The manifested neuroprotective effects on damaged neuroblastoma SH-SY5Y cells of the two compounds may be due to their inhibitory activity on Aβ aggregation and protection against oxidative stress by decreasing the ROS and increasing the mitochondrial membrane potential. Our further perspectives also deal with the structure–activity relationship studies of racemic mixtures and the design of other chromone analogues. In addition, non-mitochondrial cytoprotective mechanisms, use of other microglial cells [37], and an in vivo assessment are significant considerations to evaluate their potential as therapeutic agents against AD.

## 5. Conclusions

Chromones are heterocyclic and pharmacologically active molecules existing as natural products or synthetic compounds. The current study reports the first biological activity of the synthetic chromones diaportheone A1 and diaportheone A2. Their neuroprotective potentials were systematically evaluated in vitro using a single viability assay and their capacity to inhibit the Aβ aggregation was determined by ThT assay and molecular docking. Hence, this study provided promising scaffolds for anti-AD drug development research.

## Figures and Tables

**Table 1:** Results of the Thioflavin-T (ThT) assay on Diaportheones A1 and A2.

	% Inhibition of Aβ_1-42_ Aggregation ^a^
Diaportheone A1 (5 μM)	34.75% ± 2.5 *
Diaportheone A1 (50 μM)	80.41% ± 1.40 *
Diaportheone A2 (5 μM)	35.21% ± 2.80 *
Diapotheone A2 (50 μM)	73.68% ± 1.70 *
Phenol Red (50 μM) (Positive control)	65.78% ± 2.97

^a^ The values are expressed as the mean ± SD (*n* = 3). * Significant difference (*p* < 0.05) with the positive control using one-way ANOVA.

**Table 2:** Docking-Predicted Binding Affinities (kcal/mol) for the Aβ templates 2BEG and 2MXU. For the 2BEG template: black—site 1, red—site 2, green—site 3.

Model	2MXU		2BEG	
	Diaportheone A1	Diaportheone A2	Diaportheone A1	Diaportheone A2
1	−8.0	−7.9	−10.3	−11.0
2	−5.8	-	−8.7	−8.6
3	−6.8	−6.6	−9.7	−10.1
4	−8.3	−8.5	−6.8	−6.6
5	−6.6	−6.2	−8.8	−8.9
6	−7.3	−6.9	−10.4	−9.7
7	-	-	−8.3 −8.1	−8.4 −7.4
8	−7.1	−7.1	−7.8 −10.7	−7.6 −10.8
9	−7.5	−7.2	−9.1	−8.8
10	−7.4	−7.4	−8.8	−8.6

## Data Availability

The data presented in this study are available on request from the corresponding author. The data are not publicly available due to privacy restrictions.
